# Transcriptional Insights Suggest Altered Ripening Progression and Sugar Regulation in Japanese Indigenous Wine Grape *Vitis* sp. cv. Koshu

**DOI:** 10.3390/ijms27021061

**Published:** 2026-01-21

**Authors:** Nao Hayashi, Shunji Suzuki

**Affiliations:** Laboratory of Fruit Genetic Engineering, The Institute of Enology and Viticulture, University of Yamanashi, Kofu 400-0005, Japan; g23diaa1@yamanashi.ac.jp

**Keywords:** berry size, Koshu, ripening, sugar accumulation, transcriptome, wine grape

## Abstract

*Vitis* sp. cv. Koshu is an important Japanese indigenous wine grape cultivar. However, it possesses challenging traits for winemaking, including large berries and low sugar content. To explore the transcriptional profile associated with these characteristics, we conducted a comparative transcriptome analysis of the berry flesh of Koshu and Chardonnay at 10 days after the onset of véraison. A total of 5534 differentially expressed genes were identified, revealing a distinct transcriptional profile in Koshu. The expression patterns in Koshu suggest an altered ripening progression, characterized by the marked downregulation of the ripening marker *Grape Ripening-Induced Protein 22-like* (*GRIP22-like*) and the upregulation of photosynthesis-related genes. Despite its large-berry phenotype, cell wall-loosening genes were suppressed in Koshu, leading to the hypothesis that its size may reflect cell division in the early growth stage rather than cell expansion during ripening. Its low-sugar phenotype appears to be associated with multiple factors, including the suppression of key sugar accumulation enzyme genes, such as *sucrose synthase 2* (*SS2*) and *sucrose-phosphate synthase 1* (*SPS1*), the upregulation of *Early Response to Dehydration 6-like* (*ERD6-like*) genes, which encode putative vacuolar glucose exporters, and the suppression of cell wall-loosening genes, suggesting a potential biophysical limitation on sugar storage. This study provides the first detailed transcriptomic resource for Koshu berry and identifies key candidate genes for future breeding strategies to improve this unique cultivar.

## 1. Introduction

Grapes (*Vitis* spp.) are among the most economically important fruit crops worldwide, with around 50% of global production used for winemaking [1]. There are more than 10,000 grape cultivars, which can be broadly categorized into international and indigenous cultivars [2]. Whereas international grape cultivars are widely cultivated and recognized for their standardized production and consistent quality, this dominance has also raised concerns, including stylistic homogenization and increased susceptibility to disease and environmental stress [3,4]. In this context, indigenous cultivars are receiving increased attention as valuable resources for future viticulture. Adapted to local climates and soils, and closely associated with cultural traditions, indigenous cultivars represent a significant source of genetic diversity, which is crucial for creating novel wine styles and breeding grapevines resilient to climate change [5,6].

Koshu is a Japanese indigenous grape cultivar that has been cultivated for over 1000 years. It is currently utilized primarily for white wine production and is the most widely grown wine grape cultivar in Japan. It is the first Japanese indigenous cultivar to be listed in the International List of Vine Varieties and their Synonyms by the International Organization of Vine and Wine (OIV). The international recognition of Koshu wines has increased significantly in recent years, resulting in gold medal awards at international competitions. Considered to be a hybrid of 75% *V. vinifera* L. and 25% wild species *V. davidii* (Rom. Caill.) Foëx [7], Koshu has distinct characteristics uncommon in European international cultivars. At maturity, the berry skin develops a grayish-pink color, and the berries contain 3-mercaptohexan-1-ol (3MH) precursors, which contribute to the characteristic citrus aroma of Koshu wines [8].

Despite these unique and positive attributes, Koshu exhibits certain disadvantageous traits for winemaking, including low sugar content and large berry size. The level of soluble sugars is a major determinant of berry quality because sugars in the juice are converted into alcohol during the wine fermentation process, thereby determining the alcohol content of the wine. Compared to typical wine grapes, which have a total soluble solids (TSS) content of 20 to 24 Brix, Koshu grapes have a TSS content of 14 to 18 Brix [9]. To compensate for the low sugar content, Koshu winemakers commonly practice chaptalization, artificially adding sugar to the juice. However, this dilutes berry-derived components and negatively affects wine flavor.

In addition to sugar content, berry size is another key trait influencing berry and wine quality. The concentration of key quality components, including aroma compounds, is highest in the berry skin [10]. Consequently, a large berry size is disadvantageous as it reduces the skin-to-flesh ratio and dilutes these essential compounds. Furthermore, a negative correlation between berry size and sugar content has been reported in various grape cultivars [11,12,13]. This is highly relevant to Koshu as its berry size is 1.5 to 2 times that of European cultivars [14,15], suggesting a potential association between its characteristic large berry size and inherently low sugar content. Therefore, understanding the molecular mechanisms controlling these characteristics requires consideration of the fundamental processes of grape berry development.

Previous molecular biological studies have progressively unveiled the genetic background and physiological characteristics of Koshu. These include investigating its genetic origin through single-nucleotide polymorphism (SNP) genotyping [7], characterizing its unique genomic features via whole-genome resequencing [16], and analyzing the transcriptome of its leaves, berry skins, and internodes using both microarray and RNA-seq technologies [17,18]. However, research on the transcriptome of Koshu berry flesh, the tissue responsible for sugar storage and berry size determination, remains unexplored. This lack of information has limited our understanding of the molecular basis of Koshu berry traits.

This study aims to gain transcriptional insights into the unique berry traits of Koshu through RNA-seq-based transcriptome analysis. Grape berry ripening begins with a critical developmental transition known as véraison. This ripening stage is crucial for determining final berry quality because it regulates key events like sugar accumulation and berry softening [19]. Focusing on this critical period, we compared the transcriptome of Koshu with that of Chardonnay, one of the most widely cultivated white wine cultivars worldwide, to explore the background of its large berry size and low sugar content.

## 2. Results

### 2.1. Phenotypic Evaluation of Berry Traits

To characterize the phenotypic differences between Chardonnay and Koshu grapes, we evaluated several berry traits at two developmental stages: 10 days after the onset of véraison (DAV) and at harvest.

**Figure 1:**
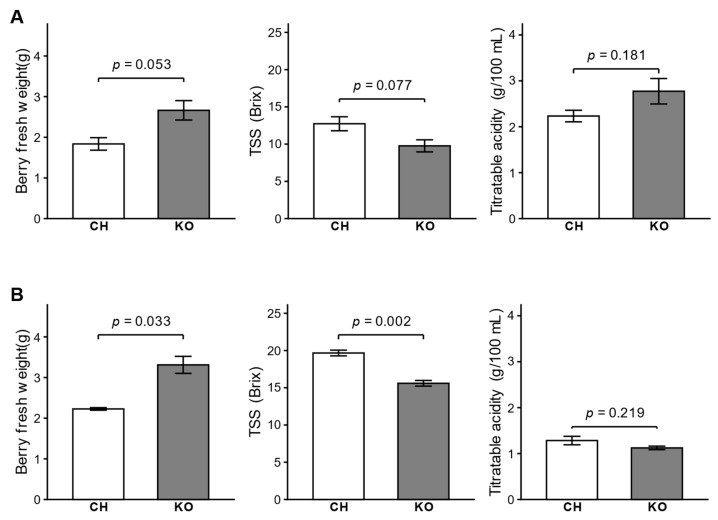
Berry traits of Chardonnay and Koshu at two developmental stages. Comparison of berry traits between Chardonnay (CH) and Koshu (KO) at (**A**) 10 days after the onset of véraison and (**B**) harvest. Within each panel, berry fresh weight, total soluble solids (TSS), and titratable acidity were evaluated. Data are presented as the mean ± standard error of three biological replicates. Statistical significance between the two cultivars was assessed using Welch’s *t*-test, and the corresponding *p*-values are shown.

At 10 DAV, Koshu had larger berries and lower sugar content than Chardonnay, although no statistically significant differences were observed between the two cultivars (*n* = 3; Figure 1A). The fresh weight of Koshu berries was approximately 1.4 times higher than that of Chardonnay berries (*p* = 0.053). TSS content was slightly lower in Koshu than in Chardonnay (*p* = 0.077). Titratable acidity levels were comparable between the two cultivars (*p* = 0.181).

At harvest, the differences in berry traits became more distinct and statistically significant (Figure 1B). The fresh weight of Koshu berries was significantly higher than that of Chardonnay berries, confirming its large-berry phenotype. The TSS content of Koshu was considerably lower than that of Chardonnay, consistent with its characteristic low-sugar trait. No significant difference in titratable acidity was found between the two cultivars.

### 2.2. Overview of Transcriptome Sequencing and Read Mapping

To investigate the molecular basis for the observed phenotypic differences, we performed RNA-seq analysis on berry flesh from both cultivars at 10 DAV. A total of 324 million paired-end reads were generated. After quality control and trimming, 322 million high-quality clean reads were retained for subsequent analysis (Table S1). On average, each sample yielded 53.7 million clean reads. The clean reads were mapped to the *Vitis vinifera* PN40024.v4 reference genome [20]. For Chardonnay, the mapping rates were consistently high, with an average of 94.6%. Uniquely mapped reads accounted for an average of 91.6%, and 5.4% of reads remained unmapped. For Koshu, the mapping rates showed variability among the biological replicates, with an average of 73.9%. The proportion of uniquely mapped reads averaged 72.8%. This variation may reflect sequence divergence associated with the *V. davidii* ancestry of Koshu. Detailed statistics for each sample are provided in Table S2.

**Figure 2:**
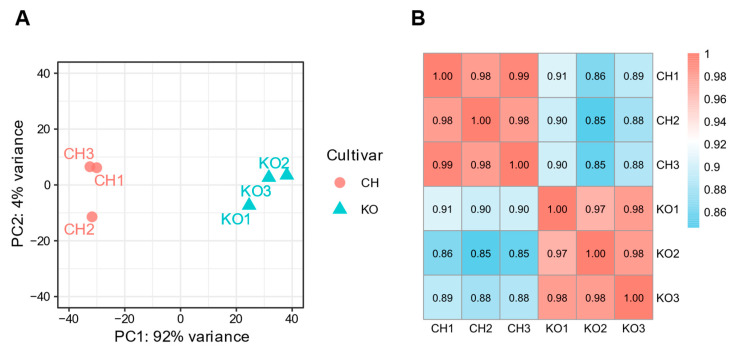
Overview of transcriptome landscape and sample relationships. (**A**) Principal component analysis (PCA) of all expressed genes. The plot was generated using variance-stabilized expression values. Each point represents an individual biological replicate of Chardonnay (CH; red circles) or Koshu (KO; blue triangles). The percentage of total variance explained by the first two principal components (PC1 and PC2) is indicated on the axes. (**B**) Heatmap of Pearson correlation coefficients among all RNA-seq samples. Correlation coefficients (*r*) were calculated from variance-stabilized expression values. The color scale indicates the degree of correlation, with red representing a high correlation (*r* = 1.0) and blue, a lower correlation.

Principal component analysis (PCA) of the expression data revealed a clear separation between the two cultivars, with the first principal component (PC1) accounting for a remarkable 92% of the total variance (Figure 2A). This indicates that the primary source of dataset variation is the difference between the cultivars. Furthermore, Pearson correlation analysis confirmed a high level of consistency among biological replicates, with strong correlations observed within the same cultivar (Figure 2B). In contrast, the correlation between the two cultivars was relatively low.

### 2.3. Differentially Expressed Gene Analysis

**Figure 3:**
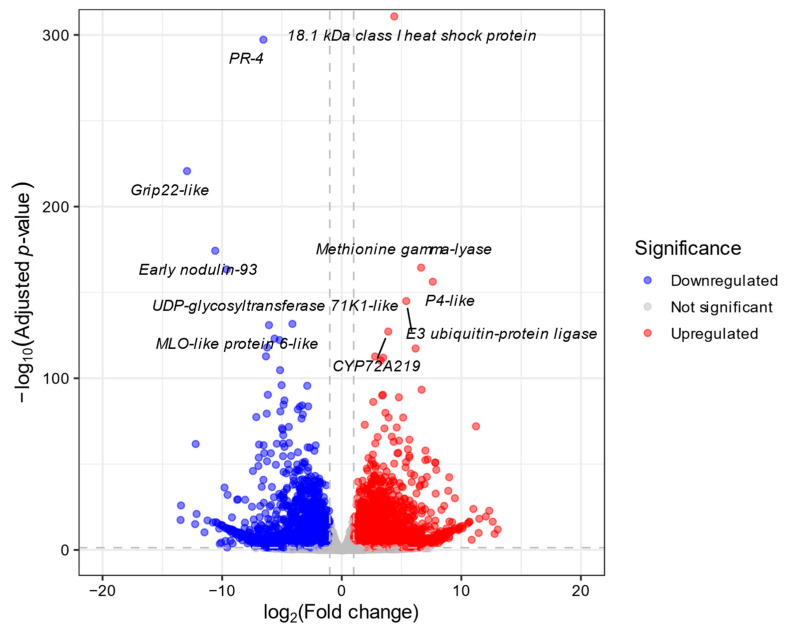
Volcano plot of differentially expressed genes (DEGs) between Koshu and Chardonnay berries at 10 days after the onset of véraison. The x-axis represents the log_2_ fold change (log_2_FC) in gene expression, and the y-axis represents the statistical significance as the negative log10 of the adjusted *p*-value. Significantly upregulated genes in Koshu (log_2_FC > 1, adjusted *p*-value < 0.05) are shown in red, whereas significantly downregulated genes (log_2_FC < −1, adjusted *p*-value < 0.05) are shown in blue. Genes with no significant expression change are shown in gray. The top five most significantly up- and downregulated genes, ranked by adjusted *p*-value, are labeled with their putative functional annotations.

Of the 35,197 annotated genes in the reference genome, 18,821 (53.5%) were expressed in either Koshu or Chardonnay. From this set, 5534 differentially expressed genes (DEGs) were identified between the two cultivars using a threshold of an adjusted *p*-value < 0.05 and an absolute log_2_ fold change (|log_2_FC|) > 1. This number represents 29.4% of the total expressed genes. Among them, 2838 genes were significantly upregulated, and 2696 genes were significantly downregulated in Koshu compared to Chardonnay (Tables S3 and S4). We further assessed the robustness of the data by applying stricter criteria (adjusted *p*-value < 0.01 and |log_2_FC| > 1.5), and, although the number of DEGs decreased under these more stringent thresholds, the main biological conclusions remained consistent. The volcano plot in Figure 3 illustrates a broad and symmetrical distribution of both up- and downregulated DEGs. Notably, among the most strongly downregulated genes was *Grape Ripening-Induced Protein* (*GRIP*) *22-like* (Vitvi06g01649). This gene was originally identified as a ripening-induced gene, the transcript levels of which were reported to increase dramatically after the onset of véraison [21]. Therefore, its significant downregulation in Koshu suggests an altered ripening progression in this cultivar. The complete lists of upregulated and downregulated DEGs are provided in Tables S3 and S4, respectively.

### 2.4. Enrichment Analysis of DEGs

**Figure 4:**
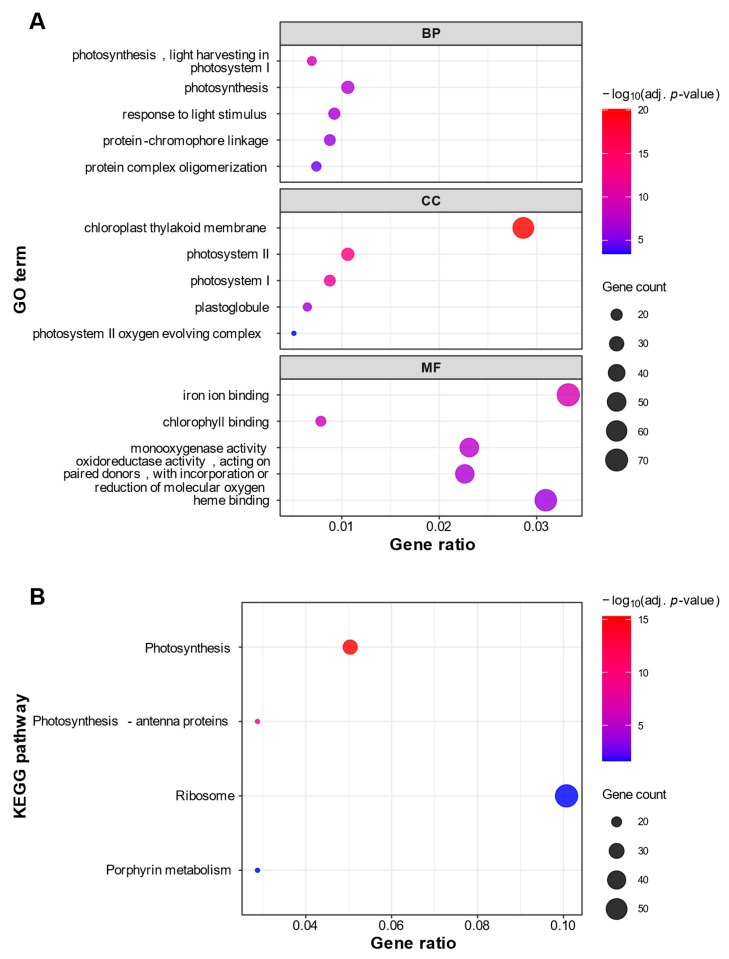
Functional enrichment analysis of genes significantly upregulated in Koshu. Dot plots of enriched (**A**) Gene Ontology (GO) terms and (**B**) Kyoto Encyclopedia of Genes and Genomes (KEGG) pathways for genes upregulated in Koshu. In the GO analysis (**A**), the plot displays up to the top five most significantly enriched terms for each of the three GO categories: Biological Process (BP), Cellular Component (CC), and Molecular Function (MF). In the KEGG analysis (**B**), the plot displays all significantly enriched pathways. For both plots, the x-axis represents the Gene Ratio (the ratio of DEGs in the term to the total number of input DEGs). The size of each dot corresponds to the number of DEGs (Gene Count) in the term, and the color corresponds to the statistical significance (−log_10_ adjusted *p*-value).

To gain insights into the biological functions of the identified DEGs, we performed Gene Ontology (GO) and Kyoto Encyclopedia of Genes and Genomes (KEGG) pathway enrichment analyses for both up- and downregulated gene sets. For the upregulated genes in Koshu, functional enrichment analysis identified 33 significantly enriched GO terms (Table S5) and 4 KEGG pathways (Table S6). Among the enriched GO terms, 12 were classified as Biological Process (BP), 9 as Cellular Component (CC), and 12 as Molecular Function (MF). Across all categories, the analysis consistently revealed an enrichment of terms associated with photosynthesis, such as ‘photosynthesis (GO:0015979)’ (BP), ‘chloroplast (GO:0009507)’ (CC), and ‘chlorophyll binding (GO:0016168)’ (MF) (Figure 4A). Consistent with the GO enrichment analysis, the KEGG pathway enrichment analysis also identified ‘Photosynthesis (vvi00195)’ as the most significantly enriched pathway (Figure 4B). These results suggest that photosynthetic activity may be maintained in Koshu berries during ripening.

**Figure 5:**
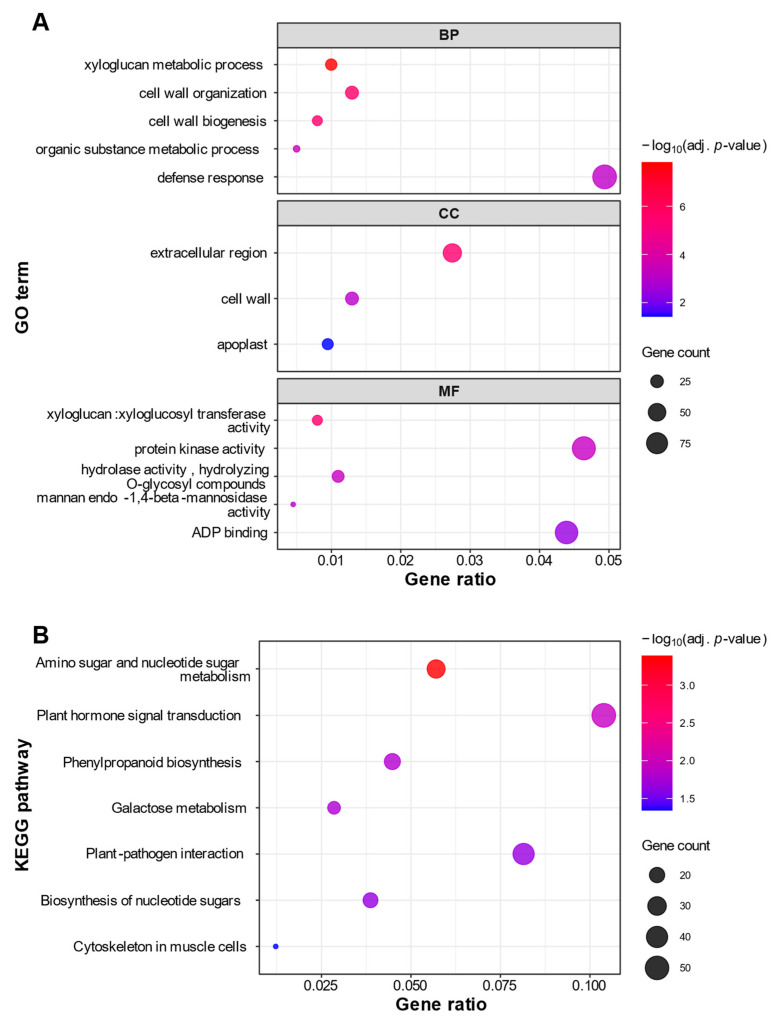
Functional enrichment analysis of genes significantly downregulated in Koshu. Dot plots of enriched (**A**) Gene Ontology (GO) terms and (**B**) Kyoto Encyclopedia of Genes and Genomes (KEGG) pathways for genes downregulated in Koshu. In the GO analysis (**A**), the plot displays up to the top five most significantly enriched terms for each of the three GO categories: Biological Process (BP), Cellular Component (CC), and Molecular Function (MF). In the KEGG analysis (**B**), the plot displays all significantly enriched pathways. For both plots, the x-axis represents the Gene Ratio (the ratio of DEGs in the term to the total number of input DEGs). The size of each dot corresponds to the number of DEGs (Gene Count) in the term, and the color corresponds to the statistical significance (−log_10_ adjusted *p*-value).

For the downregulated genes, 16 GO terms were significantly enriched, which were categorized into 6 BP, 3 CC, and 7 MF terms (Table S7). Notably, this functional enrichment of the downregulated genes revealed a clear suppression of cell wall remodeling processes. Specifically, the terms ‘cell wall organization (GO:0071555)’ and ‘xyloglucan metabolic process (GO:0010411)’ (BP) were highly enriched, alongside key enzymatic functions like ‘xyloglucan:xyloglucosyl transferase activity (GO:0016762)’ (MF), as shown in the dot plot (Figure 5A). The subsequent KEGG pathway enrichment analysis provided further evidence for the suppression of cell wall synthesis. Seven pathways were significantly enriched, including ‘amino sugar and nucleotide sugar metabolism (vvi00520)’ and ‘galactose metabolism (vvi00052)’, which provide the essential building blocks for cell wall polysaccharides (Figure 5B; Table S8).

### 2.5. Expression Analysis of Genes Involved in Sugar Accumulation

To investigate the molecular basis for the low sugar content in Koshu, we examined DEGs encoding enzymes and transporters involved in sugar accumulation, and identified 13 such genes (Table 1). Although 8 of these 13 genes were upregulated in Koshu, their absolute expression levels (transcripts per million, or TPM) were generally low. In contrast, *sucrose synthase* (*SS*) *2* (Vitvi07g00353) showed dramatically higher expression in Chardonnay than in Koshu. Similarly, *sucrose-phosphate synthase* (*SPS*) *1* (Vitvi11g00542) exhibited 3.8-fold higher expression in Chardonnay (Table 1). These high-abundance genes encode critical enzymes—SS primarily catalyzes sucrose cleavage in ripening grape berries, whereas SPS is a key regulatory enzyme in sucrose biosynthesis, whose expression level is positively correlated with sugar accumulation in grape berries [22,23].

Additionally, we identified six DEGs with negative effects on sugar accumulation, which encode sugar efflux transporters from the Early Response to Dehydration 6-like (ERD6-like) family and invertase inhibitors (Table 1). Three *ERD6-like* family genes exhibited high expression in Koshu compared to Chardonnay. Conversely, the remaining three genes were upregulated in Chardonnay, but their TPM values were generally low. These results suggest that the low-sugar phenotype in Koshu is likely influenced by a combination of two factors: the suppressed expression of high-impact, high-TPM positive regulators, such as *SS2* and *SPS1*, and the concurrent upregulation of key negative regulators.

### 2.6. Quantitative RT-PCR (qRT-PCR) Validation

**Figure 6:**
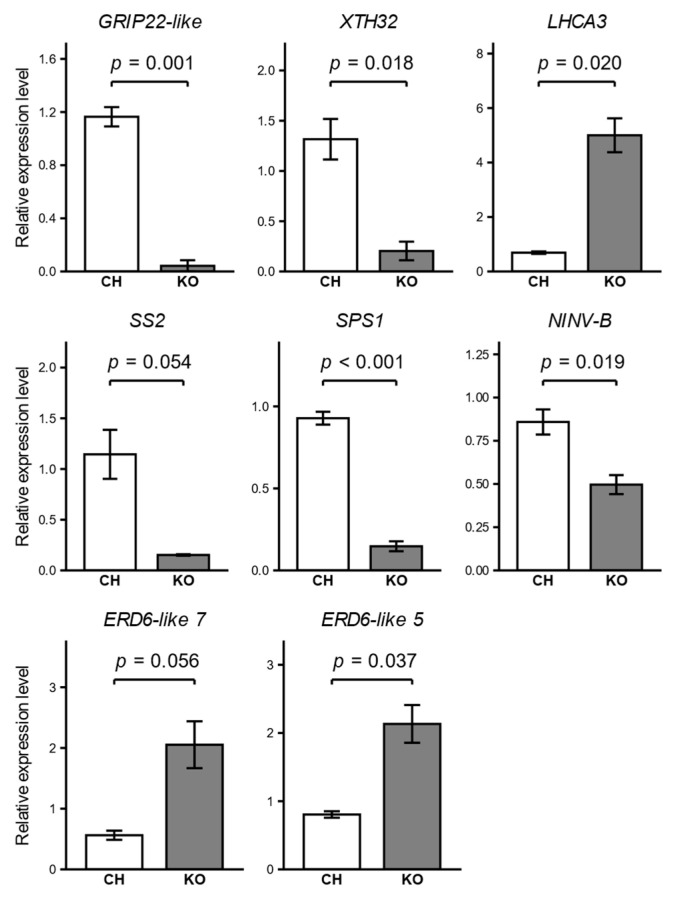
Validation of RNA-seq data by quantitative RT-PCR (qRT-PCR) analysis. Relative expression levels of eight differentially expressed genes: *Grape Ripening-Induced Protein 22-like* (*GRIP22-like*), *xyloglucan endotransglucosylase*/*hydrolase 32* (*XTH32*), *photosystem I chlorophyll a/b-binding protein 3-1* (*LHCA3*), *sucrose synthase 2* (*SS2*), *sucrose-phosphate synthase 1* (*SPS1*), *alkaline/neutral invertase B* (*NINV-B*), *Early Response to Dehydration 6-like* (*ERD6-like*) *7*, and *ERD6-like 5* in Chardonnay (CH) and Koshu (KO) berries. Gene expression was measured by qRT-PCR and is shown relative to the *VvActin* reference gene. Bars represent the mean ± standard error of three biological replicates. For the analysis of *GRIP22-like* in Koshu, one replicate was excluded as its expression was below the limit of detection. Statistical significance between the two cultivars was assessed using Welch’s *t*-test, and the corresponding *p*-values are shown.

We validated the RNA-seq results by performing qRT-PCR analysis on eight DEGs selected to represent our key findings related to ripening, cell wall remodeling, photosynthesis, and sugar accumulation, using the same RNA prepared for library construction. The relative expression patterns of these genes were consistent with the RNA-seq data (Figure 6). Specifically, the downregulation of the ripening marker *GRIP22-like* and a gene encoding xyloglucan endotransglucosylase/hydrolase (*XTH32*), as well as the upregulation of a gene encoding a photosystem I chlorophyll a/b-binding protein 3-1 (*LHCA3*), was confirmed with statistical significance between Koshu and Chardonnay. Among the genes related to sugar accumulation, the downregulation of *SPS1* and *alkaline*/*neutral invertase B* (*NINV-B*), and the upregulation of *ERD6-like 5* in Koshu berries were validated with statistical significance. For *SS2* and *ERD6-like 7*, the direction and magnitude of expression changes were also in good agreement with the RNA-seq data, although the differences were not significant (*p* = 0.054 and *p* = 0.056, respectively). Overall, these qRT-PCR results confirmed the reliability of our transcriptome analysis.

## 3. Discussion

Our comparative transcriptome analysis offers key insights into transcriptional features associated with the characteristic traits of Koshu. The data suggest an altered ripening program, reveal an unexpected suppression of cell wall remodeling genes leading to a new growth hypothesis, and identify multiple candidate molecular processes that may contribute to its low sugar content.

Before discussing these biological insights in detail, it is essential to consider the general features and potential limitations of the transcriptome data. The lower mapping rates observed in Koshu (Table S2) likely reflect its *V. davidii* ancestry, which introduces sequence divergence from the *V. vinifera* reference genome. This divergence may lead to underrepresentation of transcripts originating from more divergent genomic regions, implying that a subset of Koshu-specific alleles or lineage-specific genes could remain undetected in this analysis. Despite this limitation, the overall consistency of our comparative analysis is supported by the distinct clustering of samples by cultivar in the PCA, in which the first principal component accounted for 92% of the total variance (Figure 2A). Furthermore, a high degree of correlation was observed among the biological replicates within each cultivar (Figure 2B). These results suggest that the observed transcriptomic differences largely reflect the cultivars’ divergent genetic programs, rather than technical artifacts stemming from mapping inefficiencies. To assess the potential impact of mapping bias on the major findings, we visually inspected the read alignments of the DEGs highlighted in this study using the Integrative Genomics Viewer (version 2.19.7) [24]. Coverage and splice-junction patterns were comparable between cultivars, and no Koshu-specific read dropout or exon loss was observed. These observations suggest that the key differential expression patterns are unlikely to result from mapping artifacts, although minor effects on highly diverged or lowly expressed genes cannot be fully excluded. Future analyses using hybrid-aware or haplotype-resolved reference genomes may help further reduce these limitations.

Surprisingly, the differential expression analysis revealed that approximately 30% of all expressed genes were differentially expressed between the two cultivars, corresponding to a total of 5534 genes (Tables S3 and S4). This was considerably higher than the fewer than 250 DEGs identified in a previous transcriptome study on the leaves and internodes of Koshu and Pinot Noir [18]. This marked contrast is likely attributable to the nature of the tissue; unlike vegetative tissues, where fundamental functions remain relatively stable, the berry flesh during véraison undergoes substantial physiological and biochemical changes, such as cell wall remodeling for softening, the onset of massive sugar accumulation, and the breakdown of organic acids [10,25].

A key finding from the differential expression analysis was the marked downregulation of *GRIP22-like* (Figure 3; Table S4). Although its specific molecular function remains uncharacterized, this gene is recognized as a molecular marker for the onset of ripening, as its expression levels increase substantially after the onset of véraison [21]. Ma and Yang [26], working with the large-berried table grape cultivar Kyoho and its early-ripening mutant, reported that *GRIP22-like* was upregulated in the mutant and was associated with berry softening. Furthermore, functional enrichment analyses revealed a strong enrichment of GO terms and KEGG pathways related to photosynthesis in Koshu (Figure 4; Tables S5 and S6). This finding contrasts with the established model of grape berry ripening, which typically involves a coordinated downregulation of photosynthetic genes after véraison, accompanied by a corresponding decrease in chlorophyll content [27,28]. This general trend is supported by a study of an early-ripening grape mutant, in which photosynthesis-related pathways were significantly enriched among the downregulated genes [26]. These observations—the downregulation of a key ripening marker and the relative abundance of photosynthesis-associated transcripts—lead us to hypothesize that the ripening of Koshu follows a different molecular progression from that of small-berried European cultivars. This transcriptional pattern may be a shared characteristic among large-berried cultivars. Further investigation of other grapes, such as Muscat Bailey A, another important large-berried Japanese wine grape, and table grapes, is necessary to explore this possibility.

Grape berry enlargement is driven by two distinct mechanisms: an initial phase of rapid growth, primarily fueled by cell division, and a final phase of renewed enlargement accomplished through cell expansion during ripening [19]. Despite Koshu having significantly larger berries at harvest, functional enrichment analysis of its downregulated genes revealed a suppression of functions related to cell wall modification, including several genes encoding xyloglucan endotransglucosylase/hydrolases (XTHs) (Figure 5; Tables S7 and S8). These enzymes are crucial for loosening the cell wall, which facilitates cell expansion during ripening [29]. Although histological data on cell number and size are lacking in this study, the suppression of cell expansion-related genes in Koshu implies that its large berries may not be primarily driven by enhanced cell expansion during ripening. Instead, we hypothesize that active cell division during the initial berry growth phase could be a major contributor to the final berry size. Validating this hypothesis requires a future detailed comparative analysis of berries during the early developmental period, including histological analysis to quantify cell number and size, and expression profiling of key cell cycle regulators.

The low sugar content of Koshu is likely associated with a combination of suppressed expression of key sugar-promoting enzymes and activated expression of negative regulators (Table 1). Abundantly transcribed genes in Chardonnay, *SS2* and *SPS1*, were significantly downregulated in Koshu. *SS2* encodes a reversible enzyme that primarily catalyzes sucrose cleavage into glucose and fructose in ripening grape berries, whereas *SPS1* encodes a key sucrose synthesis enzyme thought to enhance sink strength by driving rapid sucrose turnover [22]. The importance of the *SS2* downregulation in Koshu is suggested by a study from Ren et al. [30], which reported that the same gene (Vitvi07g00353, referred to as *VvSS3* in their study) was highly expressed in a high-sugar grape cultivar. Similarly, the suppression of *SPS1* is noteworthy, given that a positive correlation between *SPS* gene expression and total sugar content has been demonstrated in Kyoho grapes [23]. Interestingly, we found that three genes encoding ERD6-like were upregulated in Koshu. Their *Arabidopsis* ortholog, *AtERDL6*, mediates the efflux of glucose from the vacuole [31,32]. *AtERDL6* overexpression decreased leaf glucose, fructose, and sucrose contents, whereas its knockout resulted in higher sugar levels in seeds [31]. It should be noted that their functions are known to be influenced by biological contexts, including cellular compartmentation and developmental stage [33], and findings from other fruit species have shown that ERD6-like proteins can participate in more complex regulatory networks involving other vacuolar sugar transport pathways [34]. The specific roles of the ERD6-like family in grapevine are still being explored; nevertheless, evidence from *Arabidopsis* suggests that the upregulation of *ERD6-like* genes in Koshu, along with the suppressed expression of sugar-promoting genes, contributes to the low-sugar phenotype by enhancing glucose efflux from the vacuole. Further functional studies will be required to clarify how ERD6-like activity integrates into the broader regulation of berry sugar accumulation.

**Figure 7:**
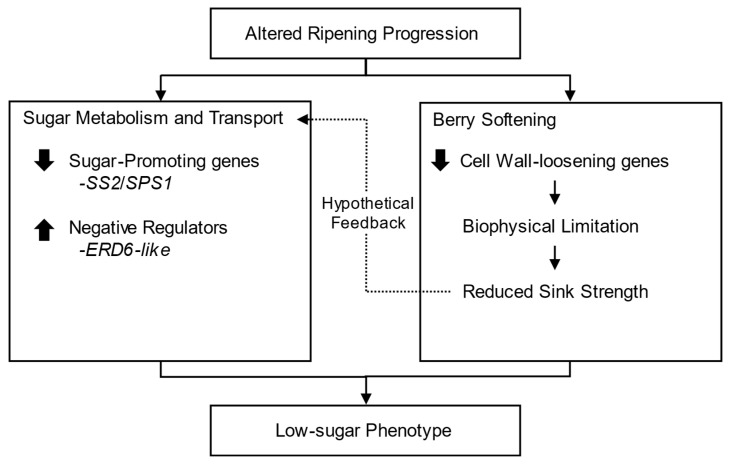
Proposed model explaining the low-sugar phenotype of Koshu berries. This model summarizes how transcriptional alterations identified at 10 days after the onset of véraison may collectively contribute to the low-sugar phenotype of Koshu. Transcriptional regulation involves the downregulation of positive regulators of sugar accumulation, including *SS2* and *SPS1*, and the upregulation of putative negative regulators such as *ERD6-like*. The broad suppression of cell wall loosening genes imposes a biophysical limitation on berry expansion, potentially leading to reduced sink strength. The dashed arrow indicates a hypothetical feedback interaction, where the reduced sink strength may further influence the transcriptional regulation of sugar metabolism and transport genes through a feedback mechanism, as suggested by previous physiological studies [35]. The thick arrows indicate the direction of differential gene expression in Koshu relative to Chardonnay (upward: upregulation; downward: downregulation).

In addition to the direct regulation of sugar-related enzymes, a biophysical mechanism may also contribute to the low sugar content in Koshu. Our analysis revealed a marked suppression of cell wall remodeling processes, suggesting insufficient berry softening. Previous studies have reported that both physical restriction of berry growth and chemically induced delays in softening can limit sugar accumulation. For instance, physically impeding berry growth resulted in slower and lower overall sugar accumulation [35]. Similarly, delaying berry softening with synthetic auxin treatments also resulted in a delayed increase in sugar content [36,37]. Therefore, it is plausible that the suppression of cell wall-loosening processes created a physical limitation on the berry’s capacity to act as a sugar sink, contributing to the low-sugar phenotype in Koshu. In addition to its potential impact on sugar import capacity, reduced sink strength resulting from limited cell wall loosening may also influence the transcriptional regulation of sugar metabolism genes in Koshu. Although speculative, such an interaction would be consistent with experimental evidence in grapevine showing that mechanical restriction of berry growth delays the transcriptional upregulation of key sugar transport and metabolism genes [35]. Taken together, as summarized in the proposed model (Figure 7), the low-sugar phenotype of Koshu is likely attributable to both transcriptional alterations in sugar-related genes—the downregulation of sugar-promoting genes and upregulation of putative negative regulators—and the suppression of cell wall loosening. Since active sugar accumulation and cell wall loosening represent the key physiological events occurring during the ripening phase [19], these observed transcriptional changes are consistent with the altered ripening progression proposed in this study.

Several limitations and future directions of this research should be acknowledged. First, our transcriptome analysis was conducted at a single time point, 10 days after the onset of véraison. Given that the transition to ripening, initiated at véraison, involves extensive transcriptional reprogramming [38,39], this stage is particularly informative for capturing cultivar-specific differences in ripening-associated processes. However, as gene expression continues to evolve dynamically throughout maturation, a time-course analysis would provide further insights into the temporal regulation of these developmental programs. Second, the mechanistic interpretations proposed for low-sugar and large-berry phenotypes of Koshu are based on transcriptomic patterns; further functional validation is necessary to confirm the causality. Lastly, since the primary differential expression and enrichment analyses highlighted genes and processes relevant to the traits of interest, we focused on characterizing these identified features. Systems-level approaches, such as co-expression network analysis or integration with genomic or QTL resources, will be valuable in future work to expand the mechanistic insights generated here.

This comparative transcriptome analysis provides an integrated view of gene expression features at the early ripening stage in Koshu, compared with Chardonnay. Specifically, we observed that Koshu exhibits a transcriptional pattern that diverges from the established ripening model, characterized by the marked downregulation of a key ripening marker, sustained expression of photosynthesis-related genes, broad suppression of cell wall-loosening genes, and the downregulation of key sugar-promoting genes together with the upregulation of putative negative regulators. These coordinated differences offer candidate molecular processes that may contribute to the characteristic low sugar content and large berry size of Koshu, while also generating testable hypotheses for future mechanistic studies.

## 4. Materials and Methods

### 4.1. Plant Materials and Sample Collection

Grapevines of *Vitis* sp. cv. Koshu and *V. vinifera* L. cv. Chardonnay were cultivated in the experimental vineyard of The Institute of Enology and Viticulture, University of Yamanashi, Kofu, Yamanashi Prefecture, Japan (35°40′ N; 138°33′ E; 273 m asl). The grapevines were approximately 30 years old, grafted onto 5BB rootstock, and trained in the Guyot style. For each cultivar, samples were collected from three individual grapevines as biological replicates at two developmental stages during the 2023 growing season. Clusters were collected between 9:00 a.m. and 11:00 a.m., 10 DAV (29 July 2023 for Chardonnay and 20 August 2023 for Koshu) and at harvest (14 September 2023 for Chardonnay and 25 September 2023 for Koshu), with one cluster randomly selected from each replicate at each stage. Immediately after collection, the clusters were subjected to trait evaluation as described below, then rapidly frozen in liquid nitrogen and stored at −80 °C until RNA extraction.

### 4.2. Berry Trait Evaluation

From each biological replicate cluster, two sets of 10 berries were collected. Each set was sampled representatively from the upper (3 berries), middle (4), and lower (3) parts of the cluster. Berries in the first set were used to determine the average fresh weight per berry. Berries in the second set were manually crushed, and the resulting juice was centrifuged at 12,000× *g* for 5 min at 25 °C. The supernatant was used to determine TSS (Brix) and titratable acidity (g/100 mL) with a refractometer (PAL-BX/ACID2, Atago, Tokyo, Japan).

### 4.3. RNA Extraction and Library Preparation

Although sampling dates differed between cultivars to ensure that all samples were collected at the same developmental stage (10 DAV), technical batch effects were minimized by performing RNA extraction and library preparation for all samples simultaneously in a single batch. Skins and seeds were removed from frozen berry samples, and approximately 100 mg of flesh tissue was excised with a surgical blade and finely chopped. The chopped tissue was placed in a microtube with a crusher and homogenized using a cryo-crusher (SK-200, Tokken, Kashiwa, Japan). The homogenized tissue was pre-treated with Fruit-mate for RNA Purification (Takara Bio, Kusatsu, Japan) to remove polyphenols and polysaccharides that interfere with RNA extraction. Total RNA was then extracted using an RNeasy Plant Mini Kit (QIAGEN, Venlo, The Netherlands) following the manufacturer’s instructions. During the extraction process, genomic DNA contamination was eliminated using the RNase-Free DNase Set (QIAGEN, Venlo, The Netherlands), as instructed by the manufacturer.

RNA quality and concentration were evaluated using NanoDrop Lite (Thermo Fisher Scientific, Waltham, MA, USA) and Agilent 2100 Bioanalyzer (Agilent Technologies, Santa Clara, CA, USA). We utilized RNA samples exhibiting an RNA Integrity Number of 8.0 or higher for subsequent analysis. RNA-seq libraries were constructed using an NEBNext Ultra RNA Library Prep Kit for Illumina (New England Biolabs, Ipswich, MA, USA). Briefly, poly(A)+ mRNA was enriched from total RNA using magnetic beads and then fragmented. First-strand cDNA was synthesized using random hexamer primers. This was followed by second-strand cDNA synthesis, which included USER enzyme digestion to ensure strand specificity. Subsequent library preparation steps included end repair, A-tailing, adapter ligation, and PCR amplification. The quality of the final libraries was verified using a Qubit fluorometer (Thermo Fisher Scientific, Waltham, MA, USA) for concentration, an Agilent bioanalyzer for size distribution, and qPCR for quantification of adapter-ligated fragments.

### 4.4. RNA Sequencing and Read Processing

Paired-end sequencing (2 × 150 bp) was performed on an Illumina NovaSeq 6000 platform (Illumina, San Diego, CA, USA). Raw sequencing data were processed using fastp (version 0.23.2) [40] with its default settings to remove adapter sequences and low-quality reads. Quality metrics, including Q20, Q30, and GC content, were assessed before and after preprocessing to ensure data integrity. The cleaned reads were then aligned to the *Vitis vinifera* PN40024.v4 reference genome [20], obtained from the Grapedia repository (https://grapedia.org/), using HISAT2 (version 2.2.1) [41]. Prior to alignment, a splice-aware index was constructed using hisat2-build with exon and splice-site information extracted from the corresponding gene annotation (v4.1). The output SAM files were converted into sorted BAM format, and alignment statistics were calculated using SAMtools (version 1.13) [42]. Raw RNA-seq data were deposited in the DDBJ Sequence Read Archive (DRA) under accession number PRJDB37650.

### 4.5. Quantification and Differential Expression Analysis

Read counts per gene were quantified using featureCounts (version 2.0.1) [43]. Reads were counted at the exon level and summarized at the transcript level. Differential expression analysis was conducted using the DESeq2 package (version 1.48.1) [44] in R (version 4.5.1) [45]. Prior to analysis, genes with a total count of less than 10 across all samples were filtered out. The count data were normalized using the median of ratios method. Statistical testing was performed using the Wald test, and *p*-values were adjusted for multiple testing using the Benjamini–Hochberg procedure. DEGs were defined as genes with an adjusted *p*-value < 0.05 and |log_2_FC| > 1. To visualize sample clustering and expression patterns, a variance stabilizing transformation (VST) was applied to the raw count data. The VST data were first used for PCA to assess sample clustering. To further assess the relationship between samples, a sample-to-sample correlation matrix was generated by calculating Pearson correlation coefficients from the VST data, and the results were presented as a heatmap using the pheatmap R package (version 1.0.13) [46]. A Volcano plot was generated using the ggplot2 R package (version 4.0.0) [47] to visualize the relationship between log_2_ fold change and statistical significance for all expressed genes.

### 4.6. Functional Enrichment Analysis

Functional enrichment analysis was performed to identify overrepresented biological functions and pathways among DEGs. GO and KEGG pathway enrichment analyses were conducted using the clusterProfiler R package (version 4.16.0) [48]. For GO enrichment analysis, DEGs were mapped to GO terms in three categories (BP, CC, and MF). An enricher function was used with a custom annotation built from the v4.1 functional annotation file obtained from Grapedia. For KEGG pathway enrichment analysis, gene identifiers were first converted into NCBI Entrez IDs using the “Gene ID Equivalences” file provided by Grapedia. The enrichKEGG function was then used with the *Vitis vinifera* organism code (“vvi”). For all enrichment analyses, the set of all expressed genes detected in this study was used as the statistical background. Gene sets with fewer than 10 or more than 500 genes were excluded from the analysis. The remaining GO terms and KEGG pathways with Benjamini–Hochberg adjusted *p*-values less than 0.05 were considered significantly enriched. Dot plots were generated using the ggplot2 R package (version 4.0.0) [47].

### 4.7. qRT-PCR Validation

To validate the reliability of the RNA-seq data, qRT-PCR analysis was performed on eight DEGs representing the key findings on ripening, cell wall remodeling, photosynthesis, and sugar accumulation. Gene-specific primers were either designed using the Primer-BLAST tool from NCBI (https://www.ncbi.nlm.nih.gov/tools/primer-blast/, accessed on 9 February 2024) or adopted from previous studies [49,50]. All primer sequences are listed in Table S9. First-strand cDNA was synthesized from the same total RNA samples used for the RNA-seq libraries using a PrimeScript RT Reagent Kit (Perfect Real Time) (Takara Bio, Kusatsu, Japan) in accordance with the manufacturer’s instructions. The qRT-PCR reactions were performed on a Thermal Cycler Dice Real Time System TP850 controlled by its accompanying software (version 5.11) (Takara Bio, Kusatsu, Japan) using TB Green Premix Ex Taq II (Tli RNaseH Plus) (Takara Bio, Kusatsu, Japan). The reaction conditions were as follows: an initial denaturation at 95 °C for 30 s, followed by 40 cycles of 95 °C for 5 s and 60 °C for 30 s. Primer specificity was verified by melting curve analysis, which showed a single peak for each amplicon. Relative expression levels were determined by generating standard curves for each gene from a five-fold serial dilution of pooled cDNA. Amplification efficiency was evaluated based on the slope of each standard curve and was confirmed to be within the acceptable range (90–110%). The quantity of each target gene was calculated from its respective curve and normalized to the reference gene, *VvActin* (Vitvi04g01613).

### 4.8. Statistical Analysis

Statistical analysis for berry trait evaluation and qRT-PCR validation was performed using R (version 4.5.1) [45]. Data are presented as the mean ± standard error of three biological replicates. Statistical differences between the two cultivars were assessed using the Welch’s *t*-test. A *p*-value less than 0.05 was considered statistically significant.

## 5. Conclusions

This study presents the first detailed transcriptome analysis of Koshu berry flesh, offering a valuable resource for understanding the molecular basis of its unique traits. Our analysis revealed that Koshu exhibits a distinct transcriptional pattern that departs from the established ripening model, offering hypotheses for understanding its characteristic traits, including low sugar content and large berry size. As the most important cultivar for Japanese winemaking, enhancing the quality of Koshu berries by overcoming their challenging traits, such as low-sugar and large-berry phenotypes, is the foremost goal. The genes highlighted in this study represent promising subjects for further functional investigation and may eventually serve as targets for future breeding and gene-editing programs aimed at increasing sugar content and reducing berry size. While it is unlikely that modifying a single gene can fully address these complex traits, identifying these key targets provides a crucial first step and a valuable roadmap for improving this unique cultivar.

## Figures and Tables

**Table 1:** Differentially expressed genes related to sugar accumulation in Chardonnay and Koshu berries at 10 days after the onset of véraison.

Gene ID	Description	log_2_FC ^1^	Adjusted *p*-Value ^2^	TPM ^3^	
				CH	KO
Genes with a Positive Role in Sugar Accumulation					
Vitvi07g00353	sucrose synthase 2	−2.67	1.08 × 10^−27^	1134.6 ± 240.5	197.0 ± 14.4
Vitvi11g00542	probable sucrose-phosphate synthase 1 isoform X1	−2.10	4.19 × 10^−12^	405.9 ± 8.1	106.4 ± 26.1
Vitvi18g00397	putative hexose transporter	2.21	3.80 × 10^−19^	37.8 ± 1.2	196.1 ± 34.2
Vitvi03g00088	probable alkaline/neutral invertase B	−1.20	1.37 × 10^−5^	24.0 ± 0.7	11.8 ± 2.1
Vitvi18g01315	sucrose transporter-like	2.50	3.92 × 10^−7^	18.1 ± 2.7	112.0 ± 46.0
Vitvi10g00679	bidirectional sugar transporter SWEET2a	1.84	3.21 × 10^−24^	7.6 ± 0.6	30.5 ± 1.2
Vitvi18g01745	putative sucrose-phosphate synthase 4	−3.48	1.54 × 10^−2^	3.2 ± 1.1	0.3 ± 0.0
Vitvi18g01215	bidirectional sugar transporter SWEET1	1.86	1.11 × 10^−2^	1.8 ± 0.6	7.2 ± 2.9
Vitvi02g00512	acid beta-fructofuranosidase	3.59	1.41 × 10^−19^	1.7 ± 0.6	22.2 ± 1.2
Vitvi18g00056	hexose transporter-like	−3.13	8.21 × 10^−5^	1.5 ± 0.1	0.2 ± 0.1
Vitvi17g00069	bidirectional sugar transporter SWEET14	2.88	1.16 × 10^−2^	1.1 ± 0.8	9.1 ± 4.4
Vitvi00g04869	acid beta-fructofuranosidase	4.32	9.77 × 10^−24^	1.1 ± 0.3	24.2 ± 2.1
Vitvi00g04570	acid beta-fructofuranosidase	5.40	3.61 × 10^−13^	0.2 ± 0.1	7.4 ± 0.9
Genes with a Potential Negative Role in Sugar Accumulation					
Vitvi07g00207	sugar transporter ERD6-like 7	1.62	4.25 × 10^−10^	26.1 ± 0.5	89.1 ± 14.4
Vitvi14g00314	sugar transporter ERD6-like 5 isoform X2	1.18	2.49 × 10^−5^	5.4 ± 0.4	13.7 ± 2.1
Vitvi14g04107	putative ERD6-like transporter	3.93	2.72 × 10^−9^	0.4 ± 0.1	6.3 ± 2.2
Vitvi04g01302	sugar transporter ERD6-like 6	−1.02	8.04 × 10^−3^	4.9 ± 0.5	2.7 ± 0.3
Vitvi15g00459	putative invertase inhibitor	−3.87	1.33 × 10^−6^	15.2 ± 4.4	1.2 ± 0.6
Vitvi05g00377	sugar transporter ERD6-like 16	−4.19	1.21 × 10^−10^	10.0 ± 3.0	0.6 ± 0.3

^1^ Log_2_-transformed fold change in expression in Koshu relative to Chardonnay. Positive values indicate higher expression in Koshu than in Chardonnay, whereas negative values indicate lower expression. ^2^ *p*-value after Benjamini–Hochberg correction for multiple testing. ^3^ Mean Transcripts Per Million (TPM) value ± standard error across three biological replicates for Chardonnay (CH) and Koshu (KO).

## Data Availability

The raw RNA-seq data presented in this study are available in the DDBJ Sequence Read Archive (DRA) under accession number PRJDB37650.

## Supplementary Materials

The following supporting information can be downloaded at: https://www.mdpi.com/article/10.3390/ijms27021061/s1.

## Abbreviations

The following abbreviations are used in this manuscript:

CH	Chardonnay
DAV	Days after the onset of véraison
KO	Koshu
TSS	Total soluble solids
